# Enterocutaneous Fistula and Pneumoretroperitoneum due to Ruptured Psoas Abscess

**DOI:** 10.1055/s-0041-1735901

**Published:** 2021-10-22

**Authors:** Tapan Patel, Shivani Patel

**Affiliations:** 1 Department of General Surgery, Medical College Baroda, Vadodara, Gujarat, India

**Keywords:** psoas abscess, enterocutaneous fistula, pneumoretroperitoneum

## Abstract

Psoas abscess is a rare condition that can present with vague clinical features. Its insidious onset can lead to a delay in diagnosis, resulting in high rates of complications and mortality. Here we describe a unique case of a patient presenting with enterocutaneous fistula and pneumoretroperitoneum due to ruptured psoas abscess.

Psoas abscess is a rare clinical finding with a reported incidence of only four cases per million population per year. 1 Psoas abscess tends to affect more male patients, with a male: female ratio of 1.62:1. 2 The reported mortality of psoas abscess is up to 19%. 3 The symptoms of psoas abscess include fever, vague flank pain, loss of appetite and weight, lump in the inguinal region, and/or limited range of movement of the hip joint. Because of nonspecific presenting symptoms, the diagnosis of psoas abscess is often delayed. The classic triad of fever, flank pain, and limited range of movement of the hip joint is present in only 30% of patients with psoas abscess. 4 Psoas abscess is classically perceived as a rare condition associated with spinal tuberculosis. However, it more likely presents secondary to gastrointestinal disease in modern surgical practice.

## Case Report

**Fig. 1:**
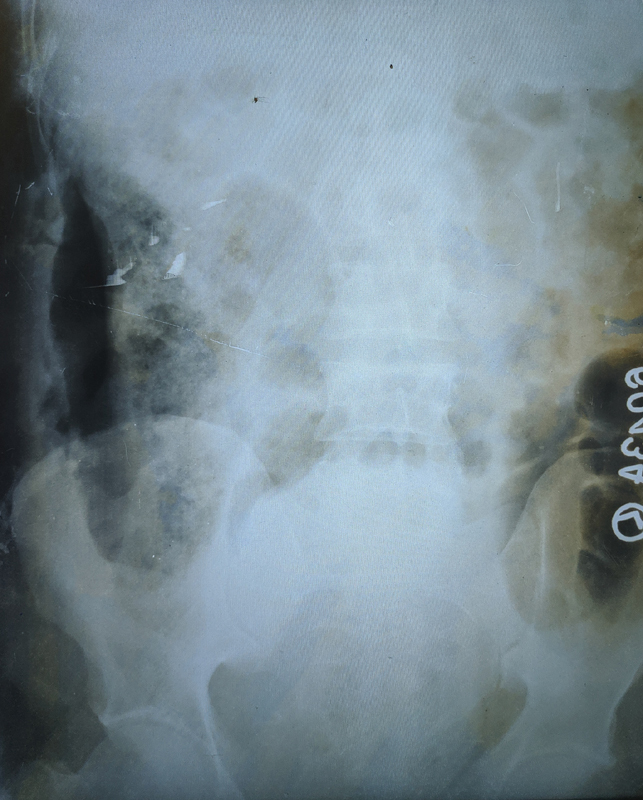
X-ray abdomen in standing position showing radiolucency in the right lateral abdomen.

A 28-year-old male presented with complaints of pain over the right lumbar region for 1 week, difficulty in walking and watery diarrhea for 3 days, and foul-smelling feculent discharge from right lumbar region for 2 days. There was no history of trauma. His temperature was 38 degrees celsius. His blood pressure, pulse, respiratory rate, and random blood sugar were in the normal range. Examination revealed a 3 cm × 2 cm opening over the right posterolateral aspect of the abdomen. Profuse, foul-smelling, feculent, greenish discharge was coming out of the opening. There was no guarding or rigidity over the abdomen. Respiratory, cardiovascular, and neurological examinations were insignificant. His total white blood cell count was 21,000 per cubic millimeter and the erythrocyte sedimentation rate was 102 after the first hour. Sonography revealed an ill-defined hypoechoic lesion of size 10 cm × 4.5 cm in the intramuscular plane of the right psoas muscle suggestive of psoas abscess. X-ray of the abdomen in standing position showed free gas in the right lateral abdomen ( Fig. 1 ).

**Fig. 2:**
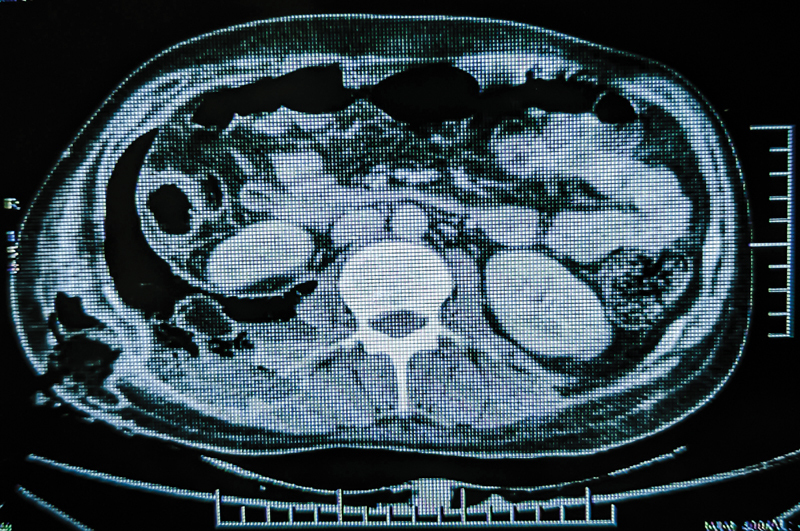
CECT abdomen showing pneumoretroperitoneum and hypodense collection on the right side. CECT, contrast-enhanced computed tomography.

**Fig. 3:**
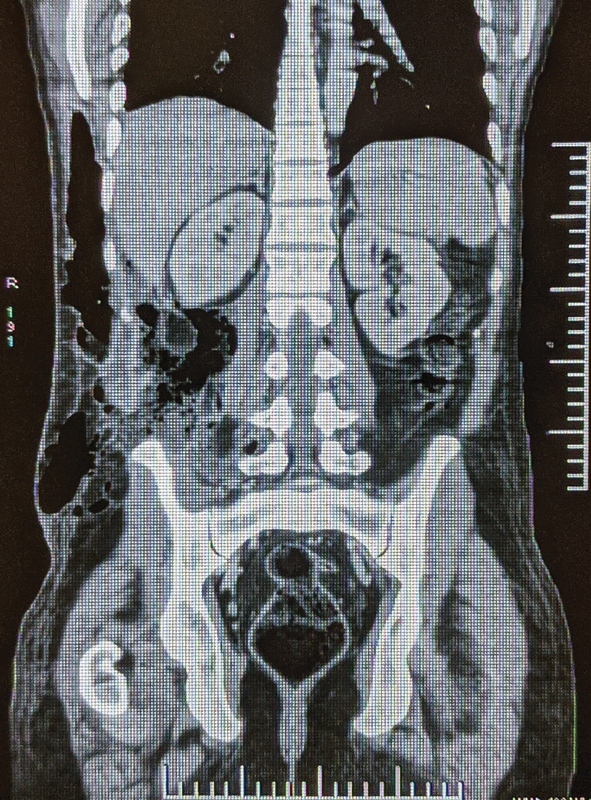
CECT abdomen ( *coronal view* ). CECT, contrast-enhanced computed tomography.

Contrast-enhanced computed tomography (CECT) was suggestive of wall enhancing hypodense collection measuring 16.7 cm × 4.3 cm × 7.5 cm in the retroperitoneum involving right psoas muscle, right erector spinae, and right quadratus lumborum muscles. Its volume was 282 cm ^3^ with air foci within it. It was seen to communicate with ascending colon through a defect measuring 7 mm × 6.7 mm. Caudally, the collection extended into the right iliacus muscle up to the lesser trochanter of the femur, at the insertion of the right iliopsoas muscle. Laterally, the collection extended into the right abdominal wall in the anterolateral and posterior aspect, the largest being 15 cm × 2.1 cm × 6.8 cm in size in the anterolateral aspect, cranially reaching up to right lower chest wall. It also extended into the subcutaneous plane and the overlying skin through a defect in the posterolateral aspect. These findings were consistent with enterocutaneous fistula ( Figs. 2 and 3 ).

Percutaneous drainage of the abscess was done. Vancomycin, metronidazole, and ceftriaxone were started empirically till the culture and sensitivity reports were obtained. Vancomycin was continued for the next 6 weeks owing to the culture reports suggestive of *Staphylococcus aureus* infection. Surgical management was done after 10 weeks by the closure of the superficial defect by debridement, local flap, and skin grafting. The management of the defect in ascending colon was done by resection of the affected part followed by end-to-end anastomosis. The patient was discharged postoperatively after 12 days. His recovery was good and the follow-up after 2 weeks, 1 month, and 6 months was uneventful. However, the patient did not consent for colonoscopy during the follow-ups.

## Discussion

Psoas abscess is classified into primary and secondary types. In the primary type, the cause is believed to be hematogenous spread from a distant site. This occurs more frequently in children, intravenous drug abusers, and immunocompromised patients. 5 *Staphylococcus aureus* is the principal bacteria involved. 6 The secondary type is due to direct extension of an intra-abdominal source of infection, mainly caused by enteric bacteria. The most common cause is Crohn's disease. 7 Other causes include appendicitis, diverticulitis, colorectal carcinoma, pyelonephritis, vertebral osteomyelitis, infected abdominal aortic aneurysms, and other rarer causes. The published literature indicates there is a difference in epidemiology between the developed and developing worlds. In Asia and Africa, 99.5% of abscesses are primary. However, in Europe, only 17.7% are primary. 6

Computed tomography (CT) is the current gold standard radiological investigation in the diagnosis of psoas abscess, with a reported sensitivity of 100%, specificity of 77%, and an accuracy of 88%. 8 9 CT can precisely portray any pathological process in the iliopsoas muscle and can reveal the related retro or intraperitoneal changes that could describe the etiology. Features of psoas abscess on CT scan are a focal low-density area within an enlarged psoas muscle. Other possible features include an abscess edge that may enhance with intravenous contrast, free gas within the lesion, and infiltration of surrounding fat. 10

Fistula is a transmural communication between two epithelialized surfaces. Enterocutaneous fistula is a tract between skin and bowel. It is usually seen in the case of Crohn's disease, diverticulitis, colon cancer, or trauma. However, our patient had no history of Crohn's disease, diverticulitis, colon cancer, or trauma. Due to the fistula, contents of the ascending colon entered the retroperitoneum. This explains the greenish-colored collection with the feculent smell. The pneumoretroperitoneum can be explained by the defect in the abdominal wall as well as the perforation in the ascending colon.

Definitive repair of the enterocutaneous fistula should be performed if spontaneous closure fails to occur by 12 weeks after nutritional optimization, control of sepsis, and wound care. Timeline to definitive repair is not firmly established but may be delayed in cases where nutrition is maintained and multiple surgeries have been previously performed. 11 Prerequisites to definitive fistula operative intervention requires nutritional optimization, sepsis control, addressing psychological morbidity, and clinical signs of softening scars and abdominal wall on examination. Avoidance and adequate repair of any enterotomy are essential as 36% of recurrent fistulas are due to inadvertent injury to the bowel. 12 Operative success rate for definitive enterocutaneous fistula resolution is 80 to 95%. 11 Recurrence rates are reduced (18%) when the involved bowel is fully mobilized and resected. Rate of recurrence is as high as 33% in case of wedge resection/bowel repair or oversewing. 13 Similarly in our case, resection of the affected part of ascending colon followed by end-to-end anastomosis was done.

## Conclusion

Psoas abscess can remain undiagnosed for a long time. It may present with significant complications like spinal nerve involvement, peritonitis, bowel perforation, abdominal aorta rupture, necrotizing fasciitis, enterocutaneous fistula, osteomyelitis, septic arthritis, and septic shock. Hence, early diagnosis and prompt treatment are necessary.

Enterocutaneous fistula following psoas abscess usually occurs secondary to underlying gastrointestinal diseases like Crohn's disease. However, it can also occur in absence of an underlying secondary pathology. It should be managed conservatively initially. Surgical management should be done only after controlling the underlying infection.
